# Retracted: Estimation of Response Functions Based on Variational Bayes Algorithm in Dynamic Images Sequences

**DOI:** 10.1155/2017/6898324

**Published:** 2017-06-20

**Authors:** BioMed Research International

BioMed Research International has retracted the article titled “Estimation of Response Functions Based on Variational Bayes Algorithm in Dynamic Images Sequences” [1]. The article was previously submitted to arXiv as: Ondřej Tichý, Václav Šmídl, “Non-parametric Bayesian Models of Response Function in Dynamic Image Sequences,” arxiv, 2015 (https://arxiv.org/abs/1503.05684).
